# Comprehensive Lung Function Assessment Does not Allow to Infer Response to Pulmonary Rehabilitation in Patients with COPD

**DOI:** 10.3390/jcm8010027

**Published:** 2018-12-27

**Authors:** Ingrid M. L. Augustin, Emiel F. M. Wouters, Sarah Houben-Wilke, Swetlana Gaffron, Daisy J. A. Janssen, Frits M. E. Franssen, Martijn A. Spruit

**Affiliations:** 1 CIRO+, center of expertise for chronic organ failure, 6085 NM Horn, The Netherlands; ewouters@ciro-horn.nl (E.F.M.W.); sarahwilke@ciro-horn.nl (S.H.-W.); daisyjanssen@ciro-horn.nl (D.J.A.J.); fritsfranssen@ciro-horn.nl (F.M.E.F.); martijnspruit@ciro-horn.nl (M.A.S.); 2 NUTRIM School of Nutrition and Translational Research in Metabolism, Maastricht University Medical Centre+, 6229 ER Maastricht, The Netherlands; 3 Department of Respiratory Medicine, Maastricht University Medical Centre+, 6229 HX Maastricht, The Netherlands; 4 Viscovery Software GmbH, 1130 Vienna, Austria; s.gaffron@viscovery.net

**Keywords:** pulmonary rehabilitation, COPD, response

## Abstract

The degree of lung function is frequently used as referral criterion for pulmonary rehabilitation. The efficacy of pulmonary rehabilitation was assessed in 518 chronic obstructive pulmonary disease (COPD) patients, after clustering based on a comprehensive pre-rehabilitation lung function assessment. Mean improvements in dyspnea, exercise performance, health status, mood status and problematic activities of daily life after pulmonary rehabilitation were mostly comparable between the seven clusters, despite significant differences in the degree of lung function. The current study demonstrates no significant relationship between the seven lung-function-based clusters and response to pulmonary rehabilitation. Therefore, baseline lung function cannot be used to identify those who will respond well to pulmonary rehabilitation, and moreover, cannot be used as a criterion for referral to pulmonary rehabilitation in patients with COPD.

## 1. Introduction

Pulmonary rehabilitation, defined as a comprehensive non-pharmacological intervention, is generally very effective in patients with chronic obstructive pulmonary disease (COPD) [1]. Indeed, statistically significant and clinically relevant improvements can be obtained for dyspnea, exercise capacity and health status compared to standard care [2,3,4].

In daily practice and in clinical trials, the degree of airflow limitation is frequently used as an indicator for referral for pulmonary rehabilitation [2]. However, not all patients with COPD with severe to very severe airflow limitation are symptomatic or limited in their daily functioning [5]. Conversely, a proportion of COPD patients with mild to moderate airflow limitation may suffer from severe dyspnea and experience everyday limitations [3]. The degree of airflow limitation, therefore, is a poor determinant of the physical and psychological status of a patient with COPD [6,7]. It has been shown that, mean improvements following exercise-based pulmonary rehabilitation are comparable after stratification for baseline airflow limitation [8,9]. Moreover, there is no difference in baseline forced expiratory volume in 1 s (FEV1) between very good and poor responders to pulmonary rehabilitation [3]. Thus, the degree of airflow limitation is a poor selection criterion for pulmonary rehabilitation. The same is true for the degree of static lung hyperinflation [10].

Recently, the heterogeneity of respiratory impairment in patients with COPD has been illustrated by the respiratory physiome, in which patients are clustered on multiple lung function attributes [11]. Whether and to what extent the respiratory physiome can be used as an indicator for referral for pulmonary rehabilitation remains currently unknown. A priori, we hypothesize that the respiratory physiome clusters are unable to infer response to pulmonary rehabilitation in patients with COPD.

## 2. Experimental Section

### 2.1. Study Design

This is an observational, prospective, single-center study about COPD, health status and cardiovascular comorbidities in relation to the outcomes of pulmonary rehabilitation (the CHANCE study) [12]. This study was approved by the Medical Ethical Committee of the Maastricht University Medical Centre+ (METC 11-3-070) and is registered as “Clinical, physiological and psychosocial determinants of the COPD Assessment Test (CAT)”, NTR 3416 [13].

### 2.2. Study Sample

Patients with COPD referred by chest physicians for a comprehensive pulmonary rehabilitation program at CIRO (Horn, the Netherlands) were included. CIRO is a third line rehabilitation center in Southern Netherlands. It specializes in offering individualized and multidimensional rehabilitation programs to patients with complex respiratory diseases. Only patients with COPD were included, and all patients gave written informed consent.

### 2.3. Measurements

**Figure 1:**
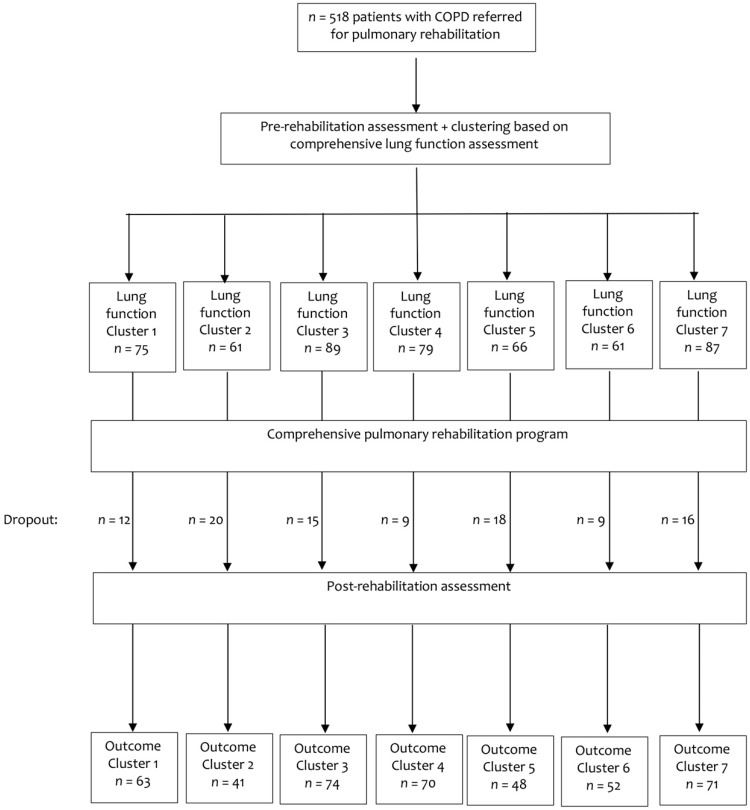
Patients before and after a 40-session comprehensive pulmonary rehabilitation program.

**Figure 2:**
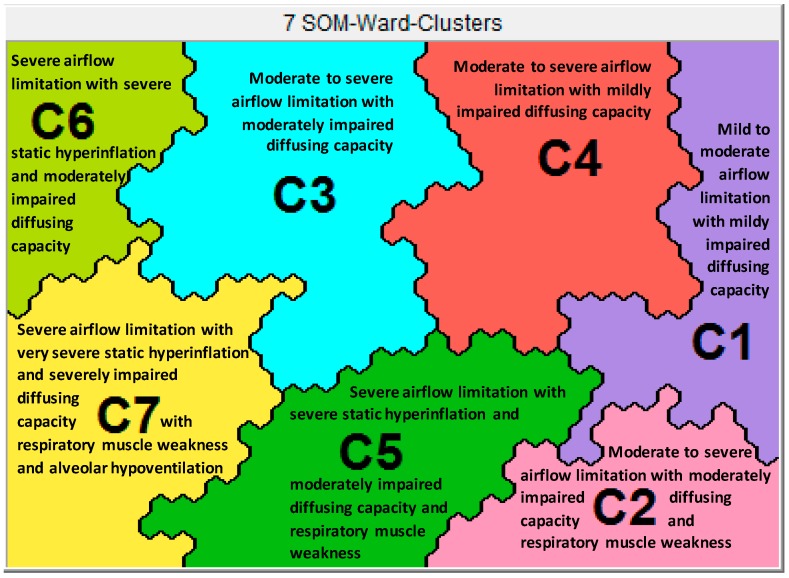
Seven different clusters of patients with COPD based on differing respiratory physiome. This figure was published in Augustin et al. [11] Legend Figure 2: The seven lung function clusters in chronic obstructive pulmonary disease (COPD) using Viscovery (Viscovery Software GmbH, Vienna, Austria). Viscovery program placed all subjects on a specific position on the map based on their profile of a comprehensive lung function assessment. Subjects with similar lung function are closer together on the map and vice versa. By drawing lines on the map, the Viscovery program could identify seven different clusters of patients with COPD with a significantly different respiratory physiome (95% confidence interval).

In total, 518 COPD patients (44% women; mean FEV1 48.6 (20% predicted); 72% stratified into group D of the Global initiative for Chronic Obstructive Lung Disease (GOLD D); mean body mass index (BMI) 26.2 (5.8 kg/m^2^)) were included. Before and after a 40-session comprehensive multidimensional pulmonary rehabilitation program, patients underwent an assessment of lung function and health status characteristics [11,12] (Figure 1). Analysis of the respiratory physiome was based on the pre-rehabilitation comprehensive lung function testing. It included post-bronchodilator spirometry to assess forced expiratory volume in 1 s (FEV1) and forced vital capacity (FVC); body-plethysmography to determine total lung capacity (TLC), residual volume (RV) and intra thoracic gas volume (ITGV); single-breath determination of carbon monoxide (TLCO); maximal static inspiratory (MIP) and expiratory mouth pressures (MEP); resting arterial partial pressure of oxygen (PaO_2_), carbon dioxide (PaCO_2_) and oxygen saturation (SO_2_). Seven different clusters of lung function impairment could be identified as described in a previous paper [11] (Figure 2). In brief, Cluster 1 had a significantly lower degree of airflow limitation, absence of static hyperinflation, and a higher diffusing capacity compared to the other clusters. Clusters 2 to 4 had similar degree of airflow limitation, but showed significant differences in static lung volumes (Cluster 3 > Cluster 4 > Cluster 2, all *p* < 0.01). Cluster 5 had a significantly lower degree of airflow limitation compared to Clusters 6 and 7 (*p* < 0.01). Static lung volumes were significantly different between Clusters 5 to 7 (Cluster 7 > Cluster 6 > Cluster 5, all *p* < 0.01). Diffusing capacity of the Lung for Carbon Monoxide (DLCO) was higher in Clusters 1, 4 and 5; lower in Clusters 3, 6, and 7, *p* < 0.01 and mouth pressures were higher in Clusters 1, 3, 4, and 6; lower in Clusters 2, 5, and 7, *p* < 0.01. Arterial blood gas values were within normal range in Clusters 1–6 [11].

The efficacy of pulmonary rehabilitation [3] was measured by the degree of dyspnea. Dyspnea was measured using the modified Medical Research Council (mMRC) scale, ranging from grade 0 (no troubles with breathlessness) to grade 4 (too breathless to leave the house). The COPD-specific version of the St George′s Respiratory Questionnaire (SGRQ-C) was also used, ranging from 0 (optimal) to 100 points (worst). A 6-min walk test (6MWT) was used to assess exercise performance. In addition, a submaximal exercise test (CWRT) was performed at 75% of the pre-determined peak work rate using an electrically braked cycle ergometer (Carefusion, Houten, the Netherlands). The Canadian Occupational Performance Measure (COPM) was used to identify specific problematic activities of daily life. Patients scored how well they were performing the problematic activities of daily life (performance score; COPM-P) and how satisfied they were with this level of performance (satisfaction score; COPM-S). Scores range between 1 (“not able to do it” or “not at all satisfied”, respectively) to 10 points (“able to do it extremely well” or “extremely satisfied”). Symptoms of anxiety and depression were measured by the Hospital Anxiety and Depression Scale (HADS) with a total score ranging from 0 (optimal) to 21 (worst) points. A score of 11 or higher indicates a severe mood disturbance.

### 2.4. Regular Intervention

The pulmonary rehabilitation program was provided in accordance with the 2013 American Thoracic Society/European Respiratory Society Statement on pulmonary rehabilitation [1], meeting the individual needs of patients with COPD [14]. The program consists of 40 sessions and can be inpatient (8 weeks, 5 days·week^−1^) or outpatient (8 weeks, 3 half days·week^−1^, followed by 8 weeks 2 half days·week^−1^). The program starts with a careful characterization of pulmonary and extra-pulmonary treatable traits in patients with COPD. From this, a patient-tailored program consisting of different treatment modules is composed. Each module consists of different interventions; physical exercise training, occupational therapy, nutritional counselling, psychosocial counselling, education and exacerbation management. Each module has a specific goal, which once achieved, contributes to the patients’ overall goal(s) of the treatment [14].

### 2.5. Statistics

All statistical analyses were performed using Viscovery Profiler 7.1 by Viscovery Software GmbH, Vienna, Austria. Information available online [15]. Self-organizing maps (SOMs, also referred to as Kohonen maps) were used to create an ordered representation of the selected attributes. The SOM method can be viewed as a nonparametric regression technique that converts multidimensional data spaces into lower dimensional abstractions. A SOM generates a nonlinear representation of the data distribution and allows the user to identify homogeneous data groups visually. Patients have been ordered by their overall similarity concerning the lung function variables measured during pre-rehabilitation assessment [11]. Using the SOM-Ward Cluster algorithm of Viscovery, a hybrid algorithm that applies the classical hierarchical method of Ward on top of the SOM topology, the seven lung function clusters have been generated [11]. Viscovery automatically identified patient characteristics that differ significantly from the average of the whole study sample using the integrated two-sided t test, with a confidence of 95% [11].

Simultaneously, the efficacy of the pulmonary rehabilitation program was evaluated for each cluster based on the minimal clinically important difference (MCID). The following MCIDs were used: −1 grade on MRC dyspnoea scale [16]; +30 m on 6-minute walk distance (6MWD) [17,18]; +100 s on cycle endurance time (CWRT [19]; +2 points on COPM-P [20]; +2 points on COPM-S [20]; −1.5 points on Hospital Anxiety and Depression Scale, Anxiety (HADS-A) [21]; −1.5 points on Hospital Anxiety and Depression Scale, Depression (HADS-D) [21]; and −4 points on St George’s Respiratory Questionnaire-Total score.

SGRQ-T [22]. For comparing outcomes of the clusters, a *p*-value of ≤0.01 was set as the level of significance.

## 3. Results

**Figure 3:**
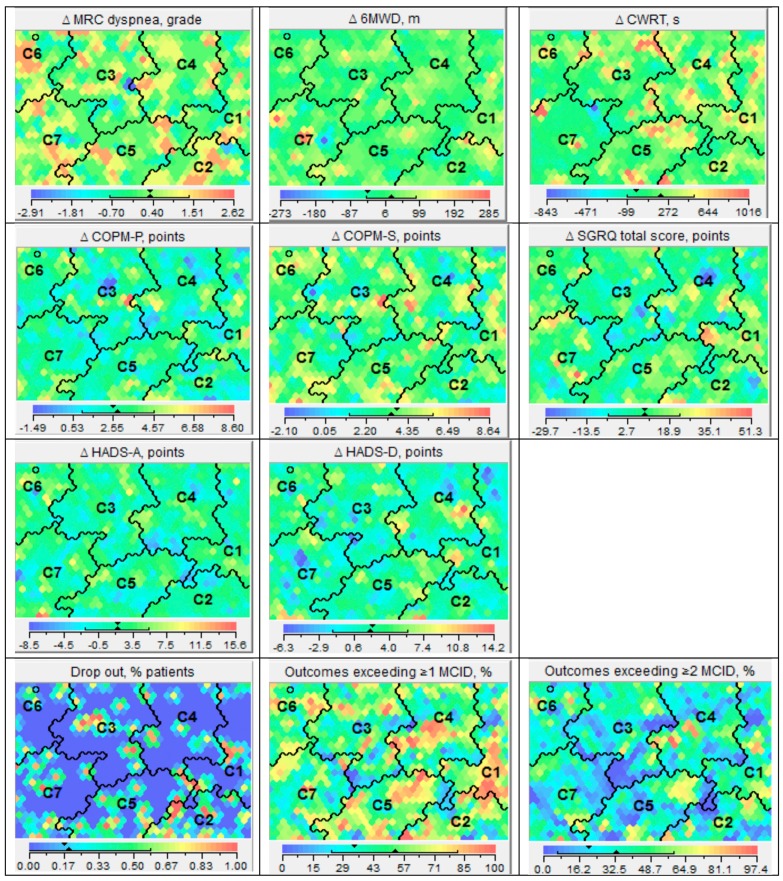
Changes following pulmonary rehabilitation. Different panels illustrating the absolute change in Medical Research Council (MRC) dyspnoea grade, 6-min walk distance (6MWD), cycle endurance time (constant work-rate test; CWRT), Canadian Occupational Performance Measure, Performance (COPM-P), Canadian Occupational Performance Measure, Satisfaction (COPM-S), Hospital Anxiety and Depression Scale, Anxiety (HADS-A), Hospital Anxiety and Depression Scale, Depression (HADS-D), and St. George’s Respiratory Questionnaire total score (SGRQ-T) for the seven lung function clusters. The other three panels demonstrate the proportion of patients not completing the pulmonary rehabilitation program, the proportion of clinically relevant outcomes (exceeding at least one minimal clinically important difference (MCID) and the proportion of clinically relevant outcomes (exceeding at least two MCID) for each lung function cluster.

A total of 419 of the 518 patients (80.9%) completed the rehabilitation program. Patients in Cluster 2 showed a significantly higher dropout rate compared to the whole sample (Figure 3). In all clusters, clinically relevant outcomes exceeding a MCID at least once were achieved. The mean improvements in the degree of breathlessness, 6-min walk distance, performance of Activities of Daily Life (ADLs), symptoms of anxiety and depression, and mean improvement in disease specific quality of life were comparable between clusters. Significant differences were only found in Cluster 2, with lower mean improvement in satisfaction with the performance of activities of daily life, and in Cluster 7, with a lower mean improvement in cycle endurance time (Table 1). Figure 3 illustrates the changes of these different outcomes per lung function cluster. Changes following pulmonary rehabilitation could not be clustered to specific physiomics profiles. Compared to the whole sample, Cluster 7 demonstrated a lower proportion of outcomes exceeding a MCID at least once.

## 4. Discussion

This is the first report on the efficacy of pulmonary rehabilitation in patients with COPD after clustering for a comprehensive lung function assessment. The results demonstrate that the degree of baseline lung function poorly predicts individual improvements in breathlessness, exercise performance, problematic activities of daily living, mood status and disease-specific health status following pulmonary rehabilitation. Even in those with the most severe respiratory impairment (i.e., Clusters 6 and 7), clinically relevant improvements were achieved. Nevertheless, one-third of the patients in Cluster 2 did not complete the program. Why patients within this cluster seem more at risk for drop-out is currently unknown and needs further evaluation.

Based on 65 randomized clinical trials involving 3822 patients for inclusion in the meta-analysis, McCarthy and colleagues concluded that pulmonary rehabilitation relieves dyspnea and fatigue, improves emotional function and enhances the sense of control that individuals have over their condition. Moreover, pulmonary rehabilitation is beneficial in improving health status and exercise capacity [2]. Our study confirms that improvements following pulmonary rehabilitation are clinically relevant and statistically significant [2,3]. According to McCarthy and colleagues, additional RCTs comparing pulmonary rehabilitation with standard COPD care are no longer warranted [2]. In order to improve outcomes, identification of markers predicting outcomes in individual patients could be very interesting. At the very least, our study illustrates that even a comprehensive lung function assessment is unhelpful in achieving this goal. Alternatively, cluster analysis could be helpful to implement specific interventions such as inspiratory muscle training in those COPD patients with respiratory muscle dysfunction but without static hyperinflation [11].

Since quality of life is determined by the degree of dyspnea, depression, anxiety and exercise performance [23], these factors should be taken into consideration in personalizing the intervention. Furthermore, as pulmonary rehabilitation programs change their emphasis towards the ability to adapt and self-manage in the face of social, physical and emotional challenges, traditional disease-related characteristics of disease severity are no longer dominant [24]. The importance of understanding the unique circumstances of the individual is now widely accepted but still neglected in pulmonary rehabilitation. The patient’s health beliefs, the way illness is approached, as well as the interactions of the patient with the medical system are affected by social, psychological, cultural, behavioral and economic factors. These unique circumstances or personomics should be considered in order to understand the patient’s preferences, values and goals [25].

Our study confirms that a comprehensive pulmonary rehabilitation program results in a heterogeneous and differential pattern of patient-related outcomes. This confirms our previous study, that a multidimensional response needs to be considered to evaluate the efficacy of pulmonary rehabilitation services [3]. Furthermore, the differential response pattern, the non-linear responses as well as the absent or poor response illustrate that a “one size fits all′′ approach is no longer applicable in pulmonary rehabilitation. In addition, non-linear responses as well as unpredictability in response must be considered as a reflection of the intrinsic complexity of the patient themselves [26].

Pulmonary rehabilitation requires multidimensional profiling of patients, not restricted to pathophysiological respiratory system involvement. Future identification of essential components of pulmonary rehabilitation should be based on a personomic perspective [25]. Comprehensive intervention can no longer be based on restoration of impairments, it needs to become person-centered.

## 5. Conclusions

The current study demonstrates no relationship between the seven lung-function-based clusters and response to pulmonary rehabilitation in patients with COPD. Therefore, baseline lung function cannot be used to identify good responders to pulmonary rehabilitation, and therefore, cannot be used as a criterion for referral to pulmonary rehabilitation in patients with COPD.

## Figures and Tables

**Table 1:** Changes following a pulmonary rehabilitation program for the seven lung function clusters.

Outcomes	Whole Sample	Cluster 1	Cluster 2	Cluster 3	Cluster 4	Cluster 5	Cluster 6	Cluster 7
All patients	*N* = 518	*N* = 75	*N* = 61	*N* = 89	*N* = 79	*N* = 66	*N* = 61	*N* = 87
Drop out, % patients	19	16	33 *	17	11	27	15	18
Number of patients completing pulmonary rehabilitation	*N* = 419	*N* = 63	*N* = 41	*N* = 74	*N* = 70	*N* = 48	*N* = 52	*N* = 71
Baseline mMRC dyspnea, grade	2.4 (1.0)	2.0 (1.0) **	2.6 (1.1)	2.1 (1.0) **	2.0 (1.0) **	2.7 (0.9)	2.8 (0.9) *	2.9 (1.0) *
ΔmMRC dyspnea, grade	−0.3 (1.1)	−0.2 (1.3)	−0.4 (1.1)	−0.2 (1.1)	−0.2 (0.9)	−0.4 (1.0)	−0.5 (1.2)	−0.4 (1.0)
• −1 grade, % patients	39	3	54	30	33	41	47	47
• −2 grades, % patients	16	16	15	16	9	21	25	11
Baseline 6MWD	424 (124)	466 (118) *	430 (131)	445 (115)	495 (94) *	413 (100)	400 (112)	340 (131) **
Δ6MWD, m	23 (67)	28 (73)	28 (71)	18 (54)	26 (60)	32 (55)	11 (52)	19 (94)
• ≥30 m, % patients	44	51	54	40	43	54	37	33
• ≥60 m, % patients	22	21	28	21	21	23	18	22
Baseline CWRT, s	296 (219)	356 (225) *	307 (297)	266 (173)	353 (221) *	293 (216)	247 (136)	242 (222)
ΔCWRT, s	206 (306)	288 (308)	189 (290)	218 (327)	254 (316	280 (305)	141 (245)	57 (265) **
• ≥100 s, % patients	52	67	50	47	61	69 *	49	23 **
• ≥200 s, % patients	36	49	32	32	49	42	36	13 **
Baseline COPM-P, points	3.9 (1.4)	3.8 (1.4)	4.1 (1.4)	4.0 (1.5)	4.3 (1.3) *	3.8 (1.0)	3.7 (1.4)	3.4 (1.4) **
ΔCOPM-P, points	2.8 (1.8)	3.1 (2.0	2.5 (2.0)	2.6 (2.1)	2.4 (1.8)	3.0 (1.5)	3.0 (1.7)	2.9 (1.6)
• ≥2 points, % patients	68	77	55	62	62	77	72	72
• ≥4 points, % patients	26	35	26	25	21	30	26	23
Baseline COPM-S, points	3.3 (1.7)	3.3 (1.7)	4.0 (1.7) *	3.4 (1.6)	3.7 (1.8)	3.2 (1.3)	2.9 (1.6)	2.9 (1.6) **
ΔCOPM-S, points	3.5 (2.2)	3.7 (2.3)	2.7 (1.9) **	3.3 (2.3)	3.3 (2.3)	3.8 (1.9)	4.0 (1.8)	3.6 (2.2)
• ≥2 points, % patients	77	77	66	75	70	86	88	75
• ≥4 points, % patients	43	46	29	38	44	46	49	49
Baseline HADS-A, points	7.8 (4.5)	7.8 (4.2)	7.1 (4.6)	7.7 (4.7)	7.3 (3.5)	7.6 (4.3)	8.5 (5.0)	8.6 (5.0)
ΔHADS-A, points	−1.7 (3.7)	−2.0 (3.8)	−0.9 (2.6)	−1.5 (3.4)	−1.1 (3.9)	−1.4 (3.3)	−2.8 (3.7)	−2.2 (4.3)
• ≥−1.5 points, % patients	51	48	46	46	48	50	60	56
• ≥−3.0 points or more, % pts	39	41	29	31	34	41	51	48
Baseline HADS-D, points	7.5 (4.3)	7.2 (4.3)	7.6 (4.6)	7.0 (4.4)	6.9 (4.0)	7.9 (3.9)	7.9 (4.2)	8.3 (4.8)
ΔHADS-D, points	−2.1 (3.7)	−1.4 (3.7)	−2.2 (3.3)	−2.2 (3.6)	−2.0 (4.0)	−2.6 (3.0)	−2.6 (3.2)	−2.2 (4.5)
• ≥−1.5 points, % patients	53	41	51	52	52	69	55	54
• ≥−3.0 points, % patients	39	32	37	36	41	43	47	38
Baseline SGRQ total score, points	61 (17)	57 (21)	61 (18)	58 (17)	53 (15) **	67 (15) *	67 (15) *	67 (16) *
ΔSGRQ total score, points	−9 (14)	−12.3 (14.6)	−9.5 (14.7)	−6.1 (13.1)	−6.6 (15.6)	−10.9 (10.7)	−11.7 (11.9)	−8.8 (15.1)
• ≥−4 points, % patients	62	75	54	52	57	71	71	56
• ≥−8 points or more, % pts	51	61	49	42	49	57	57	46
Outcomes exceeding ≥1 MCID, %	56	59	53	48	52	62	55	45 **
Outcomes exceeding ≥2 MCID, %	34	37	31	29	35	36	37	29

Legend of Table 1: The efficacy of pulmonary rehabilitation based on minimal clinically important difference (MCID). Data is presented as mean (SD), unless otherwise stated. Δ: change; mMRC: modified Medical Research Council; 6 MWD: 6-min walk distance; CWRT: constant work-rate test; COPM-P: Canadian Occupational Performance Measure, performance score; COPM-S: Canadian Occupational Performance Measure, satisfaction score; HADS-A: Hospital Anxiety and Depression Scale, anxiety scores; HADS-D: Hospital Anxiety and Depression Scale, depression scores and SGRQ-T: St. George’s Respiratory Questionnaire, total score. * = a significantly higher difference compared to the whole sample (*p* < 0.01). ** = a significantly lower difference compared to the whole sample (*p* < 0.01).
